# Domain Structure Transformation and Impedance Tuning in Partially Nanocrystallized Fe-Based Microwires

**DOI:** 10.3390/s26041200

**Published:** 2026-02-12

**Authors:** Oleg Aksenov, Artem Fuks, Alexandr Aronin

**Affiliations:** Osipyan Institute of Solid State Physics, RAS, Chernogolovka 142432, Russia; artemfux@yandex.ru (A.F.); aronin@issp.ac.ru (A.A.)

**Keywords:** microwires, magnetic domain structure, magnetic force microscopy, surface domain layer, magnetoimpedance, GMI, positive magnetostriction, nanocrystallization, FINEMET

## Abstract

Fe-based amorphous microwires were studied to examine the effect of partial surface nanocrystallization on their magnetic and electrical properties. Controlled annealing was used to induce nanocrystallization within the surface layer of the metallic core. The giant magnetoimpedance (GMI) was found to increase up to 150% compared to the as-cast microwires, which correlates with variations in the electromagnetic skin depth. Magnetic force microscopy (MFM) revealed a pronounced transformation of the magnetic domain structure: inclined and zigzag domains evolved into a ring domain configuration with radially oriented magnetization. This transformation of the domain structure occurred within the same magnetic field range where the maximum impedance response was observed. These results show a strong coupling between surface nanostructuring, domain configuration, and magnetoimpedance behavior, providing insights for optimizing Fe-based microwires for use in high-sensitivity magnetic and mechanical sensors.

## 1. Introduction

Amorphous microwires are a class of soft magnetic materials that are characterized by a strong coupling between their stress–strain state and magnetic properties. On the one hand, this is due to their amorphous structure, which leads to magnetoelastic anisotropy playing a dominant role in the formation of magnetic properties. On the other hand, microwires produced by the Taylor–Ulitovsky technique exhibit a complex stress–strain state [1]. This results in the formation of a composite magnetic domain structure consisting of several concentric layers. The orientation of the magnetic moment in the surface layer depends on the sign of magnetostriction, the stress distribution on the surface, and surface defects [1,2,3,4]. The presence of strong radial anisotropy of the surface domain layer and the existence of domain structures with a radial orientation of the magnetic moments are characteristic features for microwires with positive magnetostriction. In the central part, the magnetization is oriented predominantly along the microwire axis. This domain configuration gives rise to magnetic bistability. This type of domain structure largely governs the resulting magnetic and high-frequency properties observed in microwires (ferromagnetic resonance, giant magnetoimpedance (GMI), magnetoelasticity, and high speed of domain boundary movement).

Recent studies of the domain structure of microwires with different magnetostriction values have shown that mechanical stresses result in the formation of inclined, ring, and zigzag domain structures in the surface layer [5,6,7,8]. In parallel, studies examining the influence of crystalline structure, elemental composition, and mechanical stresses on the GMI effect or domain wall motion have repeatedly demonstrated a strong correlation between these phenomena and mechanical stresses [9,10,11,12,13,14,15,16,17,18].

Since the properties of microwires are highly sensitive to mechanical stresses and external magnetic fields, numerous attempts have been made to implement these materials in practical applications [19,20,21]. Among these properties, the GMI effect is considered particularly promising. This effect is associated with a pronounced variation in the electrical impedance of a conductor. Currently, the highest values reported for microwires reach 735% for Co-based microwires and 248% for Fe-based microwires [22,23,24]. Theoretically, GMI values of up to ~10,000% have been predicted [25,26]. Reducing the gap between experimental and theoretical GMI values is one of the main challenges.

The highest GMI values are typically found in microwires with nearly zero magnetostriction, usually Co-based microwires. However, nearly zero magnetostriction can also be achieved by alternative approaches, e.g., by using amorphous–nanocrystalline alloys. For example, in the FINEMET-type alloy (Fe_73.5_Si_13.5_B_9_Nb_3_Cu_1_), it can be achieved through controlled nanocrystallization from an amorphous state. During annealing of this alloy, nanocrystals nucleate on copper clusters via a heterogeneous mechanism. Niobium slows down the growth of nanocrystals, resulting in the formation of nanocrystals with diameters of 10–15 nm. In this case, the nanocrystals precipitated in the amorphous matrix exhibit negative magnetostriction. As the volume of the amorphous matrix decreases during nanocrystallization, the magnetostriction of the material decreases. As a result, magnetostriction values of approximately 2 ppm can be achieved with a 70/30 ratio of the volumes of the amorphous and nanocrystalline phases [27]. Previous studies have examined the influence of nanocrystallization of FINEMET microwires on the magnitude of the GMI effect [24,28]. This structural transformation increases the GMI effect; however, the values obtained are far from those observed in Co-based microwires. Moreover, a clear relationship has been established between the fabrication method and the resulting GMI magnitude. Microwires produced by the Taylor–Ulitovsky technique exhibit strong compressive stresses concentrated in the surface region and tensile stresses concentrated in the central part. After cold-drawn processing, a redistribution of stress components occurs, and the region of compressive stresses increases. Different results in the evolution of the GMI effect for microwires produced by different methods suggest that mechanical stresses significantly influence the magnitude of the observed effect. In particular, it has been demonstrated in [24] that the presence of strong compressive stresses in the surface layer can accelerate nanocrystallization in this part of the microwire. At the same time, the central part of the wire can remain amorphous. The central amorphous part ensures an increase in the strength of such microwires and maintains sensitivity to mechanical deformations due to the strong magnetostriction. Moreover, enhanced magnetic permeability in the surface layer resulting from nanocrystallization leads to an increase in the magnitude of the GMI effect. This result requires further analysis with explicit consideration of the evolution of the domain structure during nanocrystallization of FINEMET microwires.

Despite numerous studies addressing either GMI or domain structure evolution, investigations that consider both phenomena simultaneously are still limited. In particular, the influence of surface nanocrystallization on the domain structure and its relationship to the high-frequency impedance of Fe-based alloy microwires have not been sufficiently explored.

The present study aimed to systematically investigate the effect of partial nanocrystallization on the impedance of FINEMET microwires, as well as the evolution of their magnetic domain structure during nanocrystallization. This will expand the possibilities for controlling the properties of microwires for the construction of next-generation sensors.

## 2. Materials and Methods

Amorphous Fe_70_Si_18_B_9_Cu_1_Nb_2_ microwires produced by the Taylor–Ulitovsky technique (ELIRI LTD) were studied. The diameter of the metallic part was 15 μm, and the thickness of the glass shell was 7.5 μm.

As shown in [28], the amorphous structure of the as-cast microwires was confirmed by X-ray diffraction data. It was also shown that after annealing at 550 °C for 1 min, the surface layer of the microwires was partially nanocrystalline, while the central part remained amorphous. The samples under study are similar to those in [28].

The hysteresis loops of the microwires were measured using a PAR VSM Model 155 vibrating sample magnetometer.

The impedance of the microwires was measured in the range from 1 MHz to 2 GHz using a vector network analyzer at a constant power of 0.1 mW. The length of all studied microwires was 10 mm. The impedance measurement procedure was described in more detail in [28].

The glass shell was removed by chemical etching in a 60% aqueous hydrofluoric acid solution. The surface quality and geometric dimensions of the microwires were determined using a Zeiss Supra 50 VP scanning electron microscope.

The magnetic domain structure of the microwires was investigated by probe microscopy using a Solver ProM system. Magnetic force measurements were performed in the semi-contact mode using MFM01 cantilevers coated with a thin CoCr film. This method was described in more detail in [5,6].

In all experiments, the magnitude of an external magnetic field was controlled using a Hall sensor with an accuracy of 0.1 Oe.

## 3. Results and Discussion

**Figure 1:**
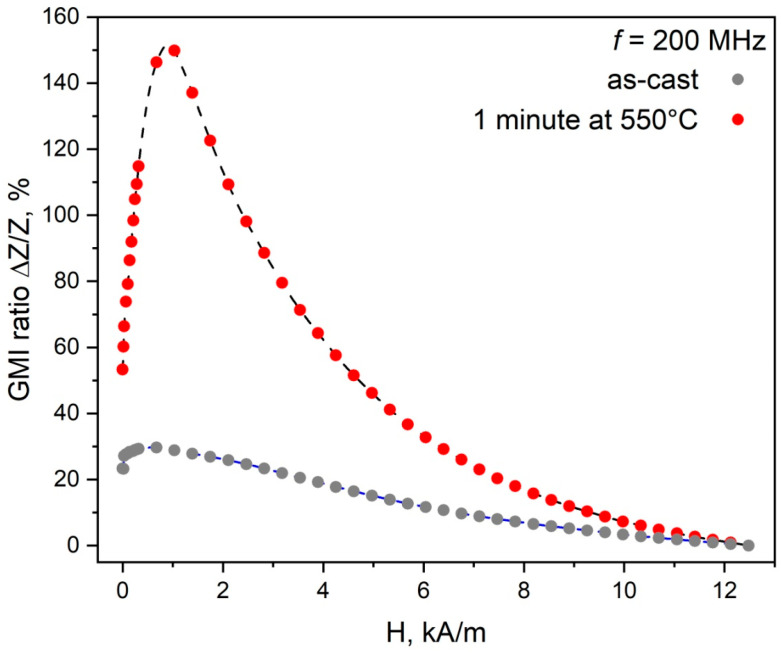
Magnitude of the GMI effect in the as-cast and annealed (550 °C, 1 min) Fe_70_Si_18_B_9_Cu_1_Nb_2_ microwires depending on the external field (current frequency of 200 MHz).

As demonstrated in [28], annealing of Fe_70_Si_18_B_9_Cu_1_Nb_2_ microwires at 550 °C for 1 min results in the formation of an amorphous–nanocrystalline surface layer and the preservation of the central amorphous part. At a current frequency of 200 MHz, the magnitude of the GMI effect for these microwires is maximum. Figure 1 shows the dependence of the GMI effect on the external magnetic field at a frequency of 200 MHz for the as-cast and annealed microwires.

As shown in Figure 1, partial nanocrystallization of the surface layer increases the GMI effect by up to 150%. This value is close to the maximum reported in the literature for FINEMET-based microwires produced by the Taylor–Ulitovsky technique. It should be noted that similar results for FINEMET microwires were previously obtained only with longer annealing times (1 h) [24]. The maximum magnetic field sensitivity is η = 0.45%/(A/m).

**Figure 2:**
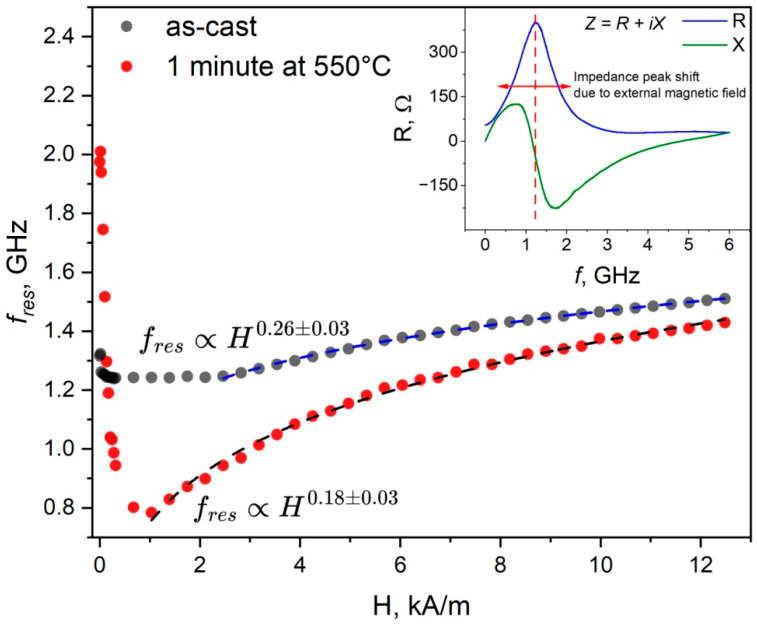
Dependence of the impedance peak frequency shift on the external magnetic field for the as-cast microwire (gray dots) and the microwire after annealing at 550 °C for 1 min (red dots).

Figure 2 depicts the dependence of the impedance peak frequency shift on changes in the external field.

The inset in Figure 2 shows typical frequency dependencies of the resistance and reactance of microwires. They demonstrate the resonant behavior of the real and imaginary impedance components: the resistance has a maximum at a certain frequency, while the reactance curve crosses zero. Such spectral behavior of the impedance components has been widely reported and is associated with ferromagnetic resonance. However, given the geometry and complex domain structure of the sample, the observed resonant response should be considered an effective high-frequency resonance arising from domain wall dynamics and magnetization rotation rather than classical ferromagnetic resonance in thin films.

The main part of Figure 2 summarizes data on resonant frequencies in various magnetic fields before and after annealing. In both cases, the behavior of the resonant frequencies clearly does not satisfy the resonance condition in thin films: $f_{r}^{2}={\left(\frac{\gamma \mu_{0}}{2\pi }\right)}^{2}\left(M_{s}+H_{eff}\right)H_{eff}$, where $f_{r}$ is the resonance frequency, γ is the gyromagnetic ratio, $H_{eff}=H_{0}+H_{K}+H_{d}$ is the effective field that is the sum of the applied magnetic field $H_{0}$, the anisotropy field $H_{K}$, and the demagnetizing factor field $H_{d}$, which is assumed to be negligibly small. In fields up to 2 kA/m, the resonance frequencies change significantly. This may result from changes in surface layer permeability associated with domain transformation during magnetization. In a field of about 1 kA/m (about 13 Oe), there is a clearly defined minimum frequency in the microwire sample after annealing. A similar non-monotonic change in the resonant frequency in an external field was previously observed in permalloy microwires in [29]. According to that paper, the anisotropy field $H_{K}$ corresponds to the magnetic field at which the minimum resonance frequency is observed. In the case of the microwire after annealing, the anisotropy field $H_{K}$ obtained from the field dependence of the resonant frequency corresponds to the field at which the maximum of the GMI effect was observed in Figure 1.

In magnetic fields above 2 kA/m, the field dependencies were approximated by power-law dependencies of the form $f=aH^{b}+c$. In strong fields, where the anisotropy field can be neglected compared to the applied field, it follows from the resonance condition that $f_{res}\propto H^{0.5}$. However, for both samples of the microwires under study, such an increase in the resonant frequency was not observed.

In the GMI regime, the current flows predominantly near the surface of the conductor at the skin depth [30]:

(1) $$\delta =\frac{1}{\sqrt{\pi \sigma \mu_{\varphi }\mu_{0}f}}=\frac{\delta_{0}}{\mu_{\varphi }^{1/2}}$$

where $\mu_{\varphi }$ is the circular magnetic permeability (relative), σ is the electrical conductivity, f is the frequency of the alternating current, and $\delta_{0}$ is the non-magnetic skin depth. In this case, the real part of the square root of the complex magnetic permeability, $Re\sqrt{\mu_{\varphi }}$, is used in the calculation:

(2) $$\mu_{\varphi }^{1/2}=\sqrt{\left|\mu_{\varphi }\right|}\cos\frac{\theta }{2}$$

where *θ* is the argument of the complex permeability. The modulus and argument of the magnetic permeability are calculated based on the real $\mu_{\varphi }^{\prime }$ and imaginary $\mu_{\varphi }^{\prime \prime }$ components following Sossmeier et al. [31]:

(3) $$\mu_{\varphi }^{\prime }=\frac{4\pi }{l}\left[\frac{\partial X}{\partial \omega }-\frac{X}{\omega }+\frac{2RX}{\omega R_{dc}}\right]$$

(4) $$\mu_{\varphi }^{\prime \prime }=\frac{4\pi }{l}\left[\frac{\partial R}{\partial \omega }-\frac{R}{\omega }+\frac{R^{2}-X^{2}}{\omega R_{dc}}\right]$$

where *l* is the length of the microwire, *R* and *X* are the resistance and reactance, respectively, and *R_dc_* is the direct current resistance of the microwire.

**Figure 3:**
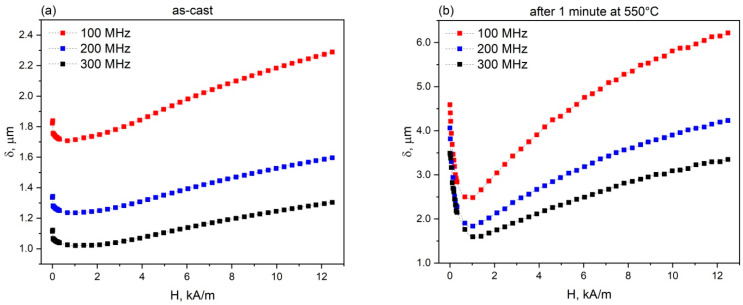
Skin depth calculated using Formula (1) for a few frequencies as a function of the magnetic field in the Fe_70_Si_18_B_9_Cu_1_Nb_2_ microwire: (**a**) as-cast and (**b**) after annealing at 550 °C for 1 min. The current frequency was 200 MHz.

Figure 3 illustrates the dependence of the skin depth at the current frequencies of 100–300 MHz on the external magnetic field in the as-cast and annealed microwires.

Figure 3 shows that the skin depth in the microwire reaches several micrometers. In the as-cast microwire, the minimum skin depth is 1.24 μm in a magnetic field of approximately 1000 A/m (~12.5 Oe). In the annealed microwire, the minimum skin depth is 1.84 μm in the same magnetic field. At a current frequency of 300 MHz, the skin depth is smaller than at 200 MHz. However, calculations of the circumferential magnetic permeability using Equations (2)–(4) show that the magnetic permeability of the microwire at 200 MHz is almost 10% higher than at 300 MHz. This may explain the impedance maximum at a current frequency of 200 Mhz.

**Figure 4:**
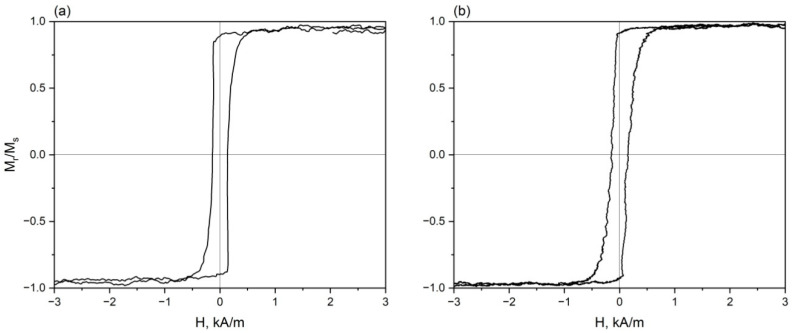
Hysteresis loops (in relative units) of the Fe_70_Si_18_B_9_Cu_1_Nb_2_ microwires: (**a**) as-cast and (**b**) after annealing at 550 °C for 1 min.

Figure 4 depicts the hysteresis loops of the Fe_70_Si_18_B_9_Cu_1_Nb_2_ microwires before and after annealing.

The thickness of the surface domain layer of a microwire can be estimated using the relationship [1]:

(5) $$t=R_{m}-R_{c}=R_{m}\left(1-\sqrt{M_{r}/M_{s}}\right)$$

where *R_m_* and *R_c_* are the radii of the microwire and the central domain layer, respectively, and *M_r_* and *M_s_* are the residual magnetization and saturation magnetization, respectively.

For the microwires under study, $\sqrt{M_{r}/M_{s}}$ for the as-cast and annealed microwires were found to be 0.94 and 0.96, respectively. Therefore, the thickness of the surface layer in the as-cast and annealed microwires was 0.45 and 0.3 μm, respectively. The minimum skin depth determined from the data in Figure 3 is several times greater than the thickness of the surface domain layer determined from the hysteresis loop. This indicates that under the GMI experimental conditions, the alternating current flows not only through the surface domain layer but also partially through the central part of the microwire with a predominantly axial orientation of the magnetic moments, thereby limiting further enhancement of the GMI effect. The highest value of the GMI effect is observed at the smallest skin depth, which may also indirectly indicate the influence of the central domain layer limiting a further increase in the GMI effect.

Furthermore, the type of domain structure in the surface layer is a significant factor. The radial orientation of the magnetic moments of the surface layer is unfavorable for achieving high GMI values (the case of the as-cast Fe-based microwires). This conclusion is supported by the experimental data; in the as-cast microwires under study, the magnitude of the GMI effect does not exceed 30%.

To evaluate the effect of the domain structure on the GMI enhancement, the surface layer was studied before and after annealing of the microwires by magnetic force microscopy.

**Figure 5:**
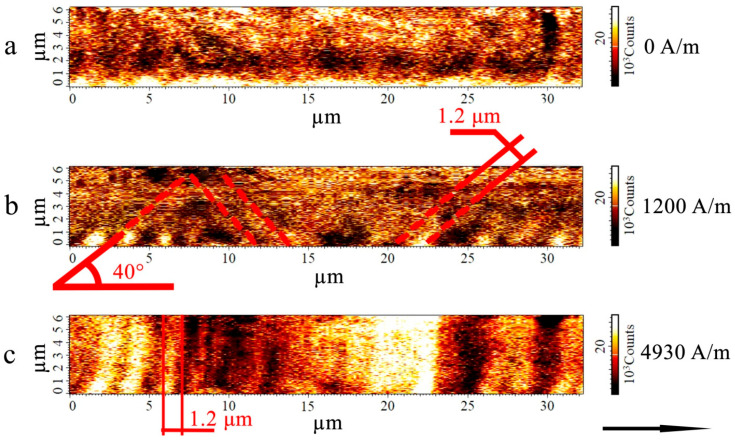
MFM images of the domain structure of the Fe_70_Si_18_B_9_Cu_1_Nb_2_ microwire. The black arrow indicates the direction of the magnetic field. The red lines denote the boundaries of individual domains in the surface layer. Images (**a**–**c**) correspond to the domain structure as the magnetic field increases. The field magnitude is indicated on the right side of each image.

Figure 5 shows magnetic force images of the domain structure of the as-cast Fe_70_Si_18_B_9_Cu_1_Nb_2_ microwire. The darker and lighter areas of the image correspond to areas with opposite orientations of the gradient of the magnetic stray fields. The direction of the cantilever magnetization is approximately perpendicular to the image plane. Under the measurement conditions used, the influence of the magnetic probe on the domain structure of the microwires is negligible. This was experimentally confirmed in our previous studies and verified in the present study. Specifically, varying the scanning height and changing the probe magnetization orientation does not lead to any qualitative changes in the observed domain structure.

As Figure 5 shows, in the absence of an external magnetic field, the phase contrast is quite weak, and the boundaries between domains with different orientations of the magnetic moment are blurred. It should be noted that MFM records the gradients of stray magnetic fields. Therefore, the resulting images reflect predominantly the change in the radial component of magnetization rather than the full vector of the magnetic moment. The observed structure exhibits a labyrinth-like pattern. The weak intensity of the phase contrast indicates a small radial component of the magnetic moment. An increase in the applied field leads to a transformation of the domain structure. In a field of 1200 A/m, there are magnetic domains inclined to the microwire axis at an angle of 40°. Domains with a shape close to zigzag, which were previously observed in microwires with positive magnetostriction in [6,8], are also present. A further increase in the magnetic field induces the formation of ring domains with a radial orientation of the magnetic moment, which correspond to the model structure for microwires with positive magnetostriction [32]. The average width of the magnetic domains (both inclined and ring) is approximately 1.2 µm. The phase contrast intensity in adjacent ring domains varies. This possibly indicates an uneven stress distribution along the length of the microwire, which leads to different values of the radial component in adjacent ring domains. As Figure 1 shows, the maximum values of the GMI effect are observed at a magnetic field of approximately 1000 A/m. It corresponds to a magnetic field at which the domain structure of the surface layer consists of inclined and zigzag domains. Ring domains are formed with a further increase in the magnetic field. Simultaneously, a decrease in the impedance of the microwire is observed.

**Figure 6:**
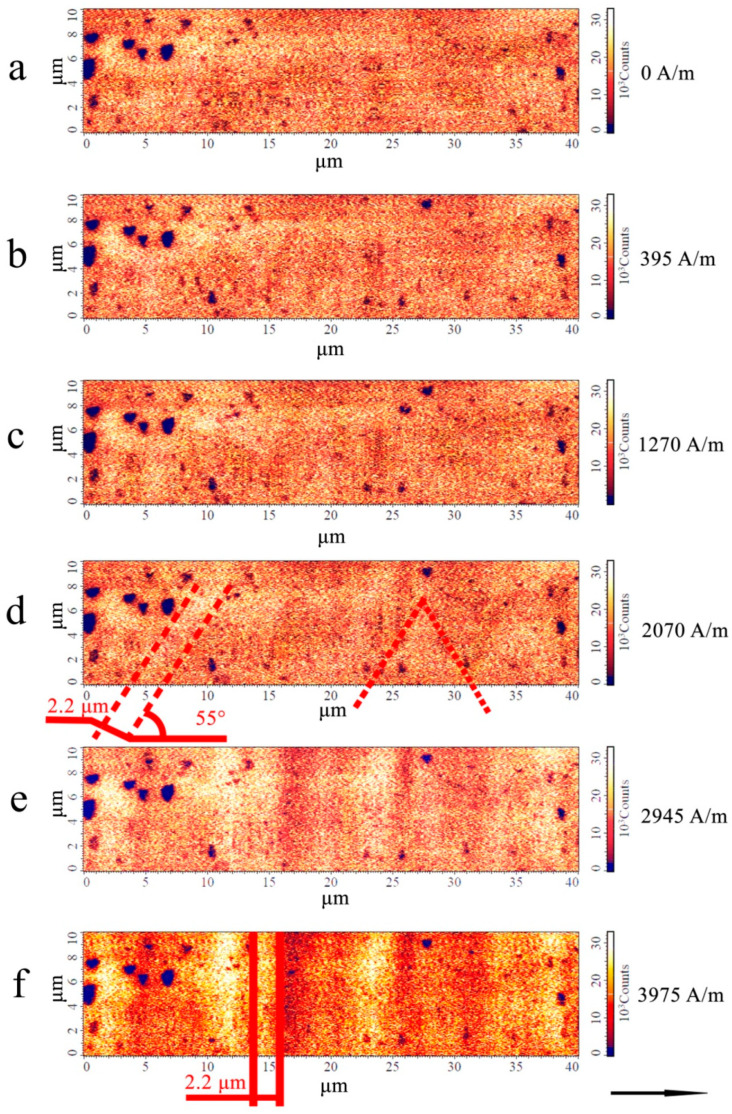
MFM images of the domain structure of the Fe_70_Si_18_B_9_Cu_1_Nb_2_ microwire after annealing at 550 °C for 1 min. The black arrow indicates the direction of the magnetic field. The red lines denote the boundaries of individual domains in the surface layer. Images (**a**–**f**) correspond to the domain structure as the magnetic field increases. The field magnitude is indicated on the right side of each image.

Figure 6 shows the magnetic force images of the microwire after annealing.

As Figure 6 shows, the intensity of the phase contrast in the microwires after annealing is significantly lower than that in the as-cast microwires (in magnetic fields close to zero). This indicates a decrease in the radial component of the magnetic moment in the domains after annealing. The change in the type of domain structure can be associated with a decrease in the magnetoelastic anisotropy constant during annealing. This is due to both stress relaxation and precipitation of the nanocrystalline phase, which leads to a decrease in the magnetostriction constant in the surface layer. It is well established that the equilibrium size of magnetic domains *D* can be related to the anisotropy constant *K* by the expression $D\sim \sqrt[{4}]{A/K}$ [33]. That is, a decrease in the anisotropy constant generally leads to an increase in the equilibrium size of magnetic domains. As Figure 5 and Figure 6 show, annealing of the microwire leads to an increase in the average domain size from 1.2 μm to 2.2 μm. At the same time, the inclination angle of the domains relative to the microwire axis also increases (from 40 to 55°). This may result from the mutual redistribution of individual stress tensor components; however, it is impossible to draw more detailed conclusions from the data obtained. Despite the weak phase contrast of the observed domain structure, inclined and individual zigzag domains are still detectable in fields below 2000 A/m.

In magnetic fields of up to approximately 2000 A/m, magnetic domains and their boundaries are barely discernible, indicating a predominantly in-plane orientation of the magnetic moments in the surface layer. Generally, this orientation of the magnetic moments correlates with higher GMI values. This is consistent with the observed increase in the GMI of up to 150% for the microwires under study.

A further increase in the magnetic field above 2000 A/m results in the gradual formation of ring domains. Ring domains in the surface layer of microwires after annealing suggest the persistence of weak positive magnetostriction in this layer. The widths of the inclined and ring domains are the same as those in the as-cast microwires.

Figure 5 and Figure 6 demonstrate that for both the as-cast and annealed microwires, the radial components of the magnetic moments of the surface layer begin to increase when an external magnetic field of more than 1000–1500 A/m is applied to the microwire. It is clear from the impedance-field dependence that the GMI effect begins to decrease as the magnetic field increases above these values. This suggests that the increase in the radial components, followed by the emergence of ring domains, leads to a reduction in the microwire impedance.

Hence, two main factors that limit the growth of the GMI effect in Fe-based microwires can be identified:

1. The small thickness of the surface domain layer causes current to flow through not only the surface layer itself but also part of the central domain layer, as well as the boundary between them.
2. When an external magnetic field is applied, the radial components of the magnetic moments in the surface layer increase, and the domain structure is transformed into a ring domain structure.

These factors can be controlled by optimizing the residual stress level in as-cast microwires (by varying the annealing parameters, glass thickness, or the manufacturing technology itself). For example, record-breaking GMI effects of 248% were obtained in [24] for FINEMET microwires produced by the cold-drawn method. It is well established that the cold-drawn method leads to an increase in compressive stress components in the central part of the wire. For example, it was shown in [32] that, compressive axial, tangential, and radial components of several hundred MPa arise in the bulk of a cylindrical sample produced by the cold-drawn method (in the part of the cylinder from 0 to 0.7 of the radius). The magnitude of these components gradually decreases and becomes positive as they approach the cylinder surface. This suggests that cold-drawn processing of microwires could have increased the thickness of the near-surface region dominated by compressive stresses. This could have led to an increase in the surface layer size. As demonstrated above, current flow through the surface and central domain layers can limit the growth of the GMI effect, with current flowing only through the surface layer being optimal. Increasing the surface layer volume could have minimized the impact of this negative effect. Together, these factors may explain the greater GMI enhancement compared to microwires produced by the Taylor–Ulitovsky technique. Moreover, such a large increase in the GMI effect in cold-drawn microwires has so far been reported only in [34], and therefore warrants further investigation.

The high-frequency impedance dependencies discussed above and the observed evolution of the magnetic domain structure suggest that the response of Fe-based microwires is determined by the combined effects of magnetic permeability, the configuration of the surface layer domains, and the distribution of the alternating current across the microwire cross-section. In this case, changes in the magnetoelastic anisotropy and domain structure resulting from both the external magnetic field and mechanical stresses directly affect the characteristics of the high-frequency response.

In practical applications, the identified patterns allow partially nanocrystallized Fe-based microwires to be considered not merely materials with an enhanced impedance response but also a tunable sensor platform capable of recording multiple physical parameters. It has been demonstrated that sensitivity, the operating range of magnetic fields, and the shape of the impedance response can be effectively controlled by manipulating the conditions under which the surface layer is formed, including the distribution of residual mechanical stresses and the degree of nanocrystallization, rather than by selecting the chemical composition. This makes these microwires promising for use in highly sensitive magnetic sensors for weak fields, current and position sensors, and multiphysical sensor systems, in which the response is determined by the combined action of magnetic fields and mechanical stresses. The ability to customize sensor characteristics allows for optimization of the operating range, increased signal stability and reproducibility, and adaptation of sensor elements to specific applications, such as structural element monitoring, non-contact measurement of magnetic fields and currents, and integration into compact and flexible sensor devices. Therefore, partially nanocrystallized Fe-based microwires provide a versatile basis for creating miniature and highly sensitive sensors, whose functional characteristics are defined during fabrication and annealing, which significantly expands their range of practical applications.

## 4. Conclusions

The effect of partial surface nanocrystallization on the magnetic domain structure and high-frequency impedance of Fe_70_Si_18_B_9_Cu_1_Nb_2_ microwires produced by the Taylor–Ulitovsky technique was experimentally studied. Controlled nanocrystallization of the surface layer was shown to increase the GMI by up to 150%, which is one of the highest values for this type of Fe-based microwire. It was found that the impedance peaked in the region of the minimum skin depth (~1.84 μm); however, the surface domain layer remained considerably thinner (0.3–0.45 μm). This led to the involvement of the central domain layer in the flow of high-frequency current and, consequently, limited further growth of the GMI effect. Magnetic force microscopy revealed that, in the magnetic field range of 1000–1500 A/m, the domain structure of the surface layer transformed from inclined and zigzag domains to ring domains with a pronounced radial orientation of magnetic moments. The onset of this transformation was found to correlate with a decrease in the impedance, indicating the crucial role of the surface layer domain configuration in the formation of the high-frequency response of the microwire.

These results allow us to identify two key factors that limit the magnitude of the GMI effect in Fe-based microwires:

1. Insufficient thickness of the surface domain layer compared to the skin depth.
2. An increase in the radial components of magnetization with an increasing external magnetic field.

These factors can be controlled through the optimization of residual mechanical stresses, annealing conditions, and microwire fabrication technology, thereby enabling broad opportunities for controlling the properties of Fe-based microwires. Possible application scenarios for microwires with a partially nanocrystalline surface layer were analyzed.

## Data Availability

The data sets generated during and/or analyzed during the current study are available from the corresponding author on reasonable request.
